# Resistance to FOXM1 inhibitors in breast cancer is accompanied by impeding ferroptosis and apoptotic cell death

**DOI:** 10.1007/s10549-024-07420-9

**Published:** 2024-07-09

**Authors:** Sandeep Kumar, Yvonne Ziegler, Blake N. Plotner, Kristen M. Flatt, Sung Hoon Kim, John A. Katzenellenbogen, Benita S. Katzenellenbogen

**Affiliations:** 1 Department of Molecular and Integrative Physiology, University of Illinois at Urbana-Champaign, Urbana, IL 61801 USA; 2 Materials Research Laboratory, University of Illinois at Urbana-Champaign, Urbana, IL 61801 USA; 3 Department of Chemistry, University of Illinois at Urbana-Champaign, Urbana, IL 61801 USA; 4 Cancer Center at Illinois, University of Illinois at Urbana-Champaign, Urbana, IL 61801 USA; 5 Institute for Genomic Biology, University of Illinois at Urbana-Champaign, Urbana, IL 61801 USA

**Keywords:** FOXM1, Estrogen receptor, Drug resistance, Proliferation, Ferroptosis, Gene expression

## Abstract

**Purpose:**

Cancer treatments often become ineffective because of acquired drug resistance. To characterize changes in breast cancer cells accompanying development of resistance to inhibitors of the oncogenic transcription factor, FOXM1, we investigated the suppression of cell death pathways, especially ferroptosis, in FOXM1 inhibitor-resistant cells. We also explored whether ferroptosis activators can synergize with FOXM1 inhibitors and can overcome FOXM1 inhibitor resistance.

**Methods:**

In estrogen receptor-positive and triple-negative breast cancer cells treated with FOXM1 inhibitor NB73 and ferroptosis activators dihydroartemisinin and JKE1674, alone and in combination, we measured suppression of cell viability, motility, and colony formation, and monitored changes in gene and protein pathway expressions and mitochondrial integrity.

**Results:**

Growth suppression of breast cancer cells by FOXM1 inhibitors is accompanied by increased cell death and alterations in mitochondrial morphology and metabolic activity. Low doses of FOXM1 inhibitor strongly synergize with ferroptosis inducers to reduce cell viability, migration, colony formation, and expression of proliferation-related genes, and increase intracellular Fe^+2^ and lipid peroxidation, markers of ferroptosis. Acquired resistance to FOXM1 inhibition is associated with increased expression of cancer stem-cell markers and proteins that repress ferroptosis, enabling cell survival and drug resistance. Notably, resistant cells are still sensitive to growth suppression by low doses of ferroptosis activators, effectively overcoming the acquired resistance.

**Conclusion:**

Delineating changes in viability and cell death pathways that can overcome drug resistance should be helpful in determining approaches that might best prevent or reverse resistance to therapeutic targeting of FOXM1 and ultimately improve patient clinical outcomes.

**Supplementary Information:**

The online version contains supplementary material available at 10.1007/s10549-024-07420-9.

## Introduction

A major problem during cancer therapy is the development of drug resistance that limits the effectiveness of long-term treatments. Therefore, there is great interest in understanding the mechanisms and cell changes that underlie resistance [1–7] and in elucidating how it might be possible to intervene to reverse or overcome resistance when it develops.

In breast cancer, and many other cancers, the oncogenic transcription factor FOXM1 is often upregulated and overexpressed, whereas it is absent or present at only very low levels in most normal adult tissues [8]. FOXM1 promotes cell cycle progression and cancer cell proliferation, tumor growth and metastasis, and high levels of FOXM1 are associated with poor patient survival [1, 8–14]. Hence, we and others have focused on the development of small molecule inhibitors of FOXM1 and have been studying how these compounds, denoted NB compounds, are able to suppress the cancer-promoting activities of FOXM1 [8–14]. We have also generated FOXM1-resistant estrogen receptor-positive and triple-negative breast cancer cells by culturing the cells continuously in increasingly high levels of NB73 for more than 6 months [13].

Cancer survival and growth, and sensitivity or resistance to therapeutic drugs, depend on the balance between proliferation and cell death programs. FOXM1 promotes breast cancer progression and invasiveness, and while it is a transcription factor well known to stimulate mitosis and proliferation, our present study shows that it also modulates cell death, with suppression of ferroptosis being very centrally involved in acquired FOXM1 inhibitor resistance and cell survival. Ferroptosis, a critical form of iron- and membrane lipid peroxidation-dependent cell death, is increasingly recognized to play crucial roles in regulating many normal and pathological cell conditions and diseases, including cancers and immune disorders [15–22].

In the current work, we examine the role of FOXM1 in promoting breast cancer cell survival and aggressiveness, and we provide information delineating how these FOXM1 inhibitors suppress FOXM1 activities in cells sensitive to them. We also describe changes that occur when breast cancer cells become resistant to the anticancer actions of the FOXM1 inhibitors. We reported previously that inhibition of FOXM1 dramatically reduced breast cancer proliferation and increased apoptosis [10, 13], effectively blocking the pro-tumorigenic and pro-metastatic actions of FOXM1 [10, 12, 13]. In the present study, we show that acquired resistance to FOXM1 inhibitor is accompanied by changes in regulators of ferroptosis and autophagic apoptosis, interrelated processes [18–22], such that cells continue to survive. Of interest, we also find that FOXM1 inhibition can synergize with activators of ferroptosis to markedly enhance anticancer activities in ER-positive and triple-negative cells, suggesting that this synergy may be broadly applicable to different subtypes of breast cancer. Further, ferroptosis activators are effective in reversing the acquired resistance to FOXM1 inhibitors. Our hope is that our findings might translate toward improving breast cancer patient treatments and clinical outcomes.

## Methods

### Cell lines and cell culture methods, FOXM1 inhibitors and cancer drugs studied

All breast cancer cell lines were obtained from the ATCC and were maintained as described [1, 23, 24]. Cells resistant to the growth-suppressive effects of inhibitors were developed by a selection of surviving cells by continuous exposure to increasing concentrations of NB73, the most potent of our NB FOXM1 inhibitors, over a period of 6 months, until a maintenance concentration of 5 µM was reached [13, 14]. All cells were tested for mycoplasma using MycoSensor PCR Assay Kit from Agilent Technologies (Santa Clara, CA). FOXM1 inhibitors were synthesized as described previously [14]. DHA (dihydroartemisinin) was from Selleckchem (Houston, TX) and JKE1674 from MedChemExpress (Monmouth Junction, NJ).

### Cell viability and synergy analyses

WST-1 assay (Roche, Basel, Switzerland) was used to quantify cell viability [14]. Assays were performed in triplicate and statistically analyzed using Graph Pad Prism 9.0. Synergy in the effectiveness of FOXM1 inhibitors and other cancer drugs in suppression of cell viability was evaluated through Zero Interaction Potency (ZIP) synergy score determinations[8], as well as Bliss and Loewe model assessments [25–28], using the Synergy Plus online tool (https://www.synergyfinderplus.org) [28].

### Gene regulation analysis by quantitative RT-PCR and RNA-seq transcriptional profiling

Total cell RNA was isolated using TRIzol (Invitrogen) and reverse transcribed using MMTV reverse transcriptase (New England BioLabs). Real-time PCR was performed using SYBRgreen PCR Master Mix (Quantabio) as described [13]. Three or four independent cell samples per condition were analyzed. Primer sequences were obtained from the Harvard Primer Bank. mRNA levels of genes were normalized to the housekeeping gene 36B4 and fold change calculated relative to vehicle-treated samples. RNA-seq analyses were as previously described [13], and genes of interest were studied further by RT-PCR.

### Western blot analyses

Whole-cell extracts were prepared using 1 × RIPA lysis buffer (Thermo Fisher, Waltham, MA) supplemented with 1 × protease and phosphatase inhibitor cocktail (Millipore Sigma, Burlington, MA). Proteins were separated on 4–12% SDS-PAGE gels and transferred to nitrocellulose membranes. All antibodies were from Cell Signaling Technology (FOXM1, Cat# 5436; ERα, Cat# 8644; LCN2, Cat# 44,058; GPX4, Cat# 52,455; SLC7A11, Cat# 12,691) except for NUPR1, which was from Proteintech (Cat# 15056-1-AP). All were used at a 1:1000 dilution except for NUPR1, used at 1:500, and β-actin, used at 1:5000 dilution as an internal loading control. IRDye 800 CW goat anti-rabbit secondary antibody (LI-COR, Cat# 926-32211) and IRDye 680 CW goat anti-mouse secondary antibody (LI-COR, Cat# 926-68070) were diluted (1:5000) for incubation with the blots. Band intensities were analyzed with LI-COR Odyssey Image Studio 5.2 software that avoids saturation, eliminates comparison of multiple exposures, and allows digital analysis of bands of all intensities, with accurate protein quantification over a broad linear range. Molecular weight markers were Chameleon Duo markers from LI-COR (8–260 kDa) or Precision Plus Dual Color Markers from BioRad (37–250 kDa).

### Transmission electron microscopy (TEM)

TEM was performed on embedded cell samples cut into 70 nm sections and imaged using a JEOL JEM-1400 TEM.

### MitoTracker analysis and evaluation of iron and lipid peroxidation

Cells were seeded (3 × 10^4^ cells/well) in 8-well chamber slides (Ibidi Co.) and cultured overnight at 37 °C in a 5% CO_2_ incubator. After 24 h, the supernatant was discarded and cells were washed with serum-free medium three times. Cells were incubated with compounds for the indicated times and washed as above. 1 µM FerroOrange (an intracellular Fe2 + ion probe, Ex: 561 nm, Em: 570–620 nm) and 1 µM Liperfluo (lipid peroxide detection probe, Ex: 488 nm, Em: 500–550 nm) working solution (from Dojindo Technologies) were added for 30 min. Cells were washed with serum-free media and then observed under a confocal fluorescence microscope (Leica Microsystems TCS SPE). Images were analyzed using Image J software. MitoTracker analysis of mitochondrial membrane potential was conducted with fluorescence intensity monitored by live confocal microscopy.

### Colony formation assays

Cells were plated in 6-well plates (1500 cells/well) in triplicate. After overnight incubation, cells were treated with compounds for 24 h and maintained at 37 °C in a 5% CO_2_ incubator for 10–14 days, with medium changes every 3 days. Colonies were fixed using 4% paraformaldehyde and stained with 0.5% crystal violet. A colony was defined as a group of at least 50 cells, and the counting was performed using Image J software.

### Wound healing/scratch assays

Cells were seeded in 6-well plates. Upon reaching confluency, a wound was scratched with wound scratcher and drugs were added immediately. Samples (*n* = 4 per condition) were monitored with an EVOS XL Core Imaging Microscope every 24 h and wound closure was determined at 72 h.

### Transwell migration assays

Assays used transwell chambers (8 μm PET membrane, Corning 3464). Cells (25 × 10^3^ per well) were treated for 24 h with compounds and then seeded in the upper chambers of a 24-well plate in 150 μl serum-free medium. The lower chambers were filled with 800 μl culture medium with 5% FBS and incubated at 37 °C for 72 h. Cells in the upper surface of the membrane were removed with a cotton swab and cells in the lower chamber were stained with Crystal Violet. Images taken with an inverted phase contrast microscope were analyzed using Image J software.

### Statistical analyses

Analysis of variance (ANOVA), 2-way ANOVA with multiple comparisons, or Student’s* t*-test, as appropriate, used GraphPad Prism 9.0 software. Significance was designated as * for *p* < 0.05, ** for *p* < 0.01, *** for *p* < 0.001, and **** for *p* < 0.0001.

## Results

### Resistance to FOXM1 inhibition and cell survival are associated with suppression of ferroptosis and autophagic cell death

**Fig. 1:**
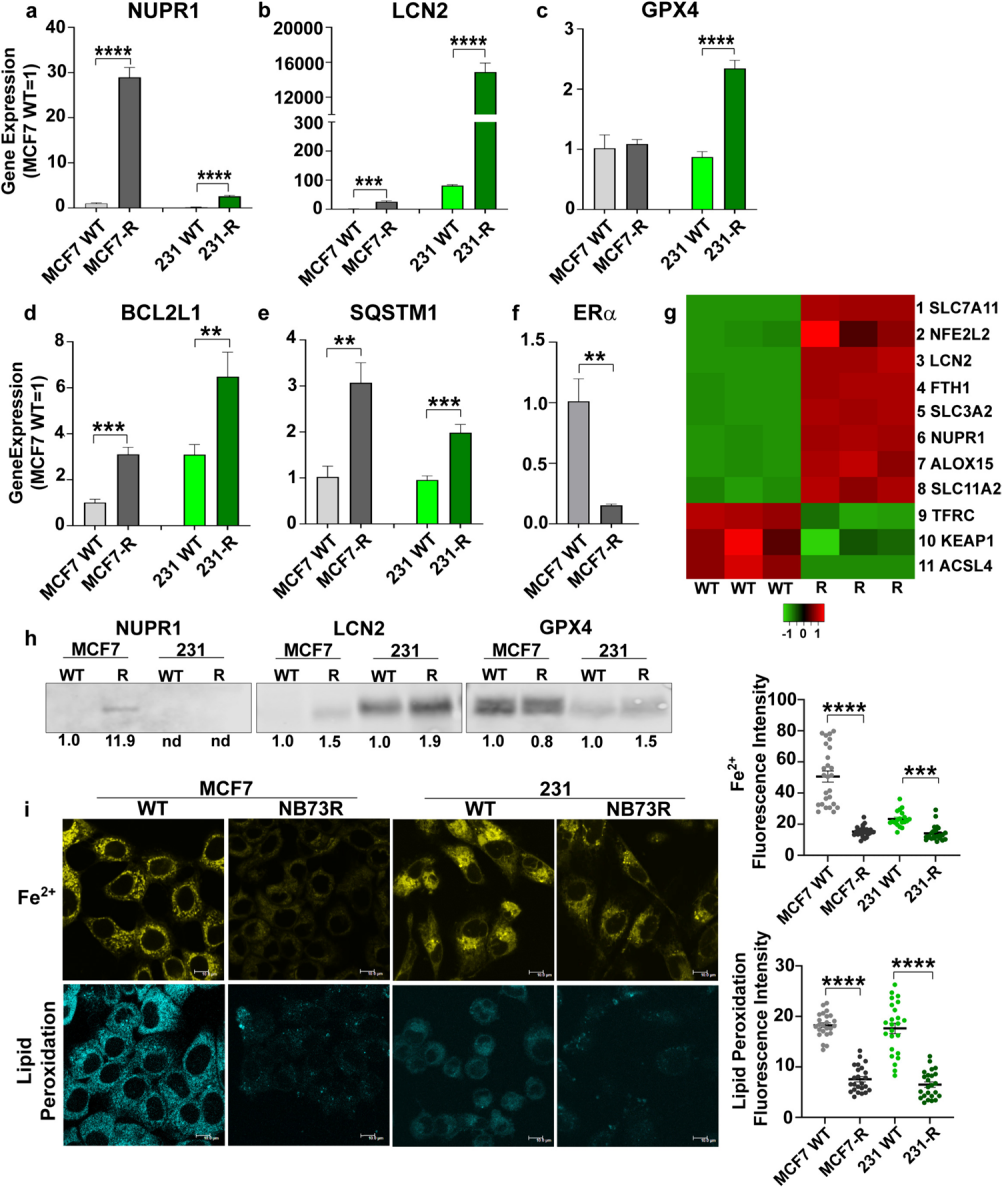
Upregulated expression of genes and proteins suppressing ferroptosis and hallmark activities associated with ferroptosis in cells that have acquired resistance to the FOXM1 inhibitor NB73. **a–f** Wild-type (WT) parental MCF7 or MDA-MB-231 cells and MCF7 or 231 cells resistant to NB73 (MCF7-R or 231-R) were assayed for expression of the indicated genes by qRT-PCR. WT cells were maintained in cell culture medium with control vehicle (0.01% DMSO) and resistant cells were maintained in cell culture medium containing 5 µM NB73. Values are mean ± SEM of 3 determinations. ERα is expressed only in the MCF7 cells, so no ERα data are shown for 231 cells in panel **f**. **g** Heat map of the expression of ferroptosis-related genes in WT and resistant (R) MCF7 cells. Scale is log twofold change. **h** Western blots showing levels of NUPR1, LCN2, and GPX4 in WT and resistant MCF7 and MDA-MB-231 cells. Numbers indicate quantitation relative to the internal loading control β-actin. nd indicates not detected. **i** Measurements of intracellular Fe^2+^ and lipid peroxidation in MCF7 WT and NB73R cells, and in 231 WT and NB73R cells. Fluorescent cell images are shown at the left (scale bar = 10 nm), with quantitation of the fluorescence intensity per cell in multiple fields shown in the graphs at the right. Stars indicate p values of *p < 0.05; **p < 0.01; ***p < 0.001; ****p < 0.0001

To elucidate cellular changes that accompany the development of resistance to FOXM1 inhibition, we compared WT parental breast cancer cells sensitive to suppression of cell viability by FOXM1 inhibitor with cells that acquired resistance to FOXM1 inhibitor NB73 by long-term (> 6 months) treatment with the drug, as reported previously [13]. Our RNA gene expression data (Fig. 1a–c) revealed that drug resistance in both ER-positive MCF7 cells and triple-negative breast cancer (TNBC) MDA-MB-231 cells was accompanied by marked upregulated expression of genes including NUPR1 (nuclear protein 1, also known as COM1, candidate of metastasis), LCN2 (lipocalin 2), and GPX4 (glutathione peroxidase 4), all considered to encode anti-ferroptotic, cell survival proteins [29–35]. Of note, the expression of two of these three ferroptosis modulating genes (NUPR1 and LCN2) was greatly increased in both NB73-resistant cell lines, with GPX4 RNA showing increase only in the 231 resistant cells.

Resistant cells also showed elevated RNA levels of anti-apoptotic BCL2L1 as well as increased expression of SQSTM1 (Sequestome 1), an autophagy receptor [36] shown to regulate activation of the NFκB signaling pathway and to be involved in selective macroautophagy (Fig. 1d, e), consistent with known interrelationships between ferroptotic, autophagic, and apoptotic pathways that enable cell survival and drug resistance. Expression of ERα, a master regulator in MCF7 cells, was greatly reduced with NB73 drug resistance (Fig. 1f), as expected [13]. Further, as seen in the heat map in Fig. 1g, resistant cells showed increased expression of 8 genes generally considered to be inhibitors of ferroptosis and reduced expression of 3 genes that usually activate ferroptosis. Also, as seen in the Western blots in Fig. 1h, resistant cells contained higher than WT levels of NUPR1, LCN2, and GPX4, consonant with alterations at their RNA levels. Thus, resistant cells appear to be rewired to block cell death and survive.

In view of the changes in genes and proteins thought to be associated with ferroptosis in NB73-resistant (NB73R) cells, we examined levels of cell ferrous iron (Fe^2+^) and lipid peroxidation, two hallmarks of ferroptosis (Fig. 1i). NB73R cells exhibited greatly reduced levels of intracellular Fe^2+^ and lipid peroxidation compared to WT parental cells, further supporting that drug-resistant cells might survive by alterations that resist ferroptotic cell death.

### NB73-resistant cells show elevated expression of cancer stem-cell markers and altered expression of FOXM1 target genes regulating proliferation

**Fig. 2:**
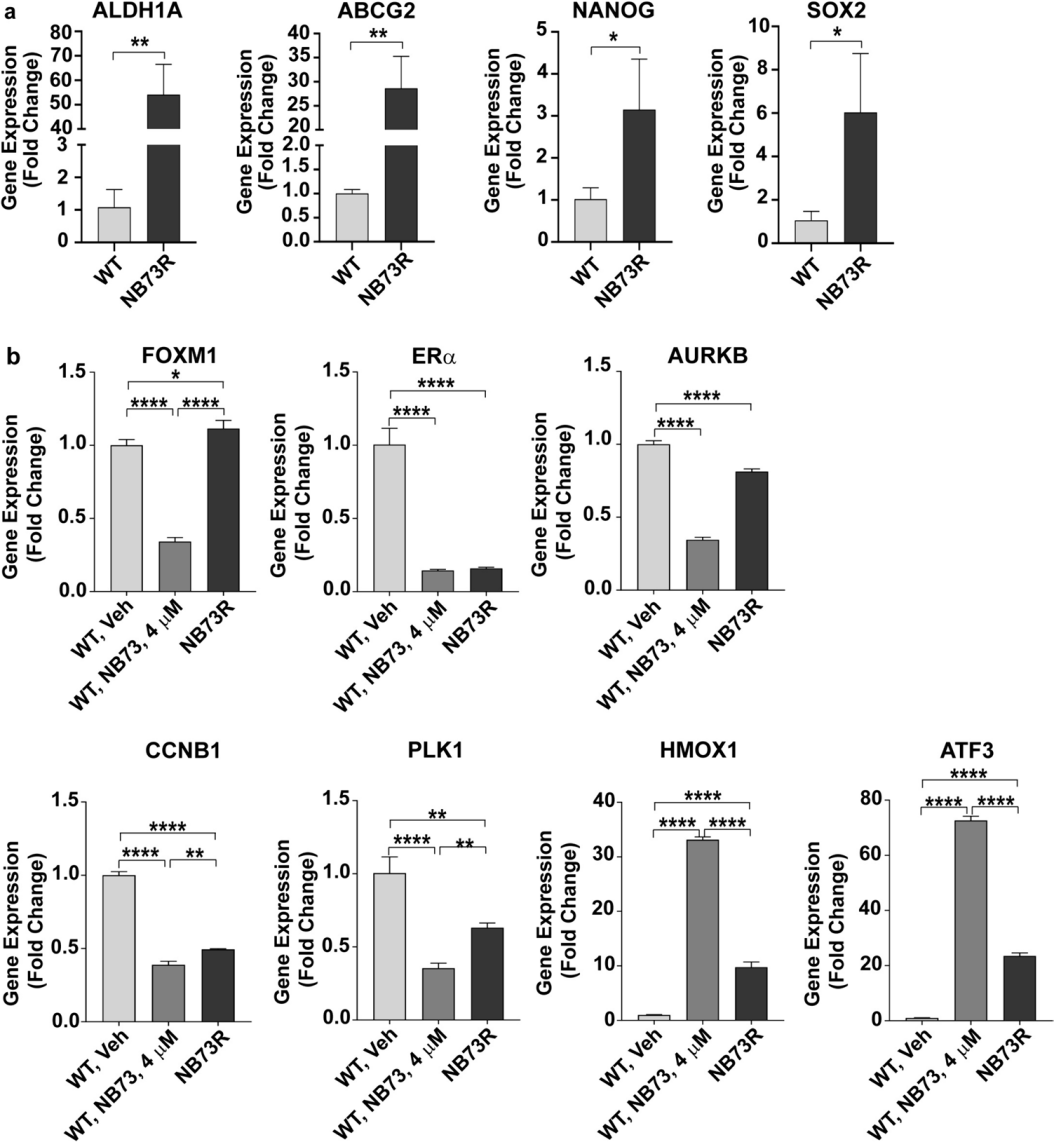
Increased stem-cell marker gene expression in NB73-resistant cells and comparison of the expression of FOXM1 and FOXM1 target genes in WT cells treated with NB73 and in 73R cells maintained long term in NB73. **a** WT or NB73R MCF7 cells were examined for expression of stem-cell markers by qRT-PCR. Values are mean ± SEM of 3 determinations and stars show p values as defined in Fig. 1 legend. **b** Inhibition of the expression of FOXM1 and FOXM1 upregulated genes (AURKB, CCNB1, PLK1), and upregulation of ATF3 and HMOX1 in WT MCF7 cells treated with 4 µM NB73 for 24 h, and recovery of some gene expressions in the 73R cells to that more resembling the WT Veh cells. RNA was extracted from cells and expression of different genes was monitored by qRT-PCR. Assays were run in triplicate. Values are mean ± SEM. *p < 0.05; **p < 0.01; ***p < 0.001, ****p < 0.0001

Cancer stem cells play a major role in the development and progression of breast cancer and in therapeutic resistance [37]. FOXM1 is often dysregulated and more highly expressed in cancer stem cells [1, 38]. Of interest, we observed that resistance to NB73 was associated with elevated expression of stem-cell marker genes ALDH1A, ABCG2, NANOG, and SOX2 (Fig. 2a), suggesting the likely importance of stemness in the development of this resistance.

Wild-type parental cells that are sensitive to NB73 inhibition showed reduced expression of the FOXM1 upregulated target genes, AURKB, CCNB1, and PLK1, and increased expression of the FOXM1 downregulated target genes, HMOX1 and ATF3, upon treatment with NB73 (Fig. 2b). Expression of FOXM1 and ERα were both reduced with NB73. By contrast, in the NB73-resistant cells maintained in a high (5 μM) concentration of NB73, expression of the proliferation-related genes returned to that more like the WT control vehicle cells, as did the expression of FOXM1, whereas ERα expression remained low in the resistant cells. Thus, NB73-sensitive cells showed gene expressions reflecting the inhibition of their viability by NB73, whereas resistant cells showed altered expression of proliferation-associated FOXM1 target genes, consistent with their continued viability despite the presence of a high concentration of FOXM1 inhibitor.

### Changes in mitochondrial morphology and metabolic activity

**Fig. 3:**
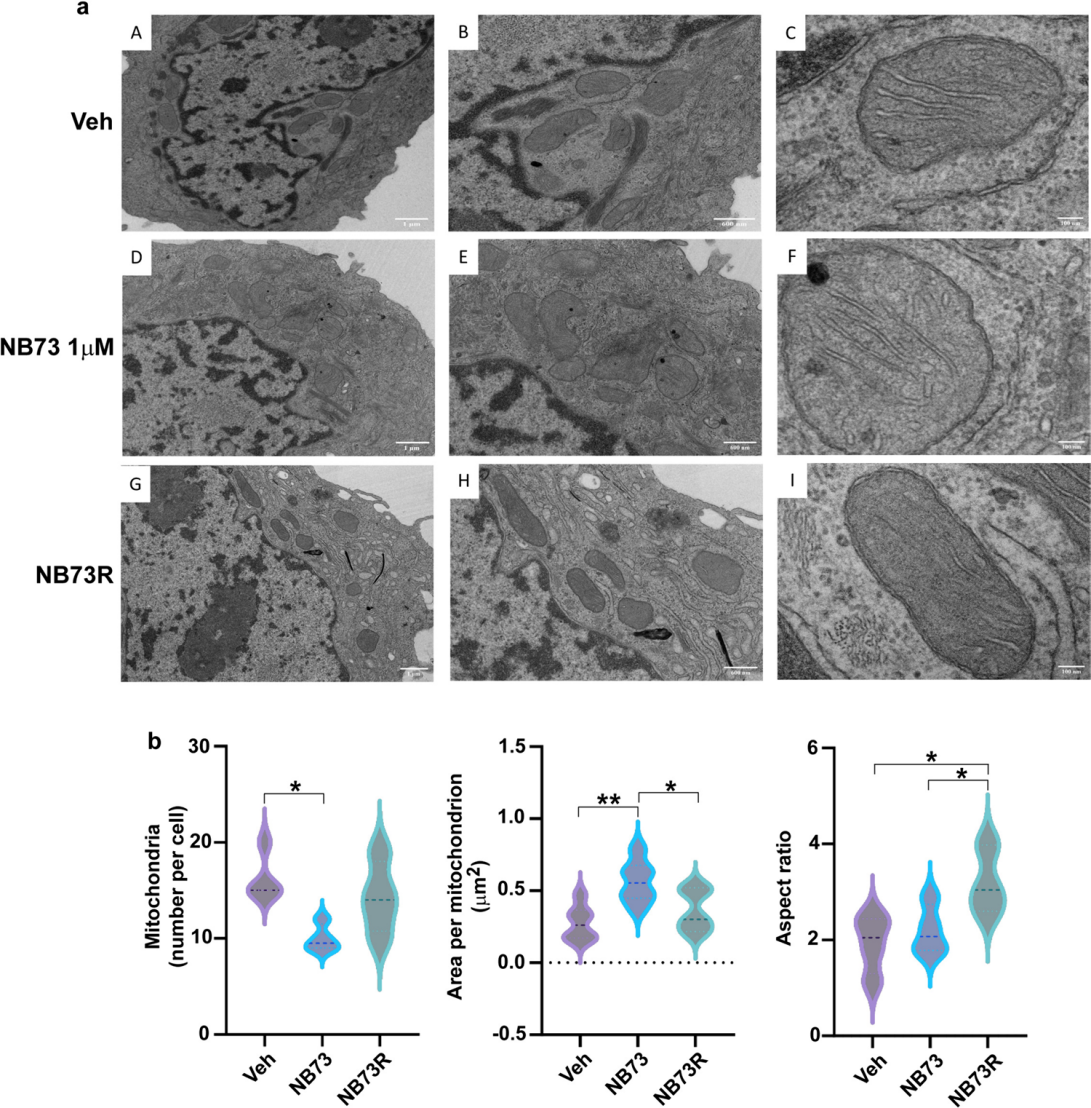
Transmission electron microscopy (TEM) analyses to monitor cell morphology and mitochondrial integrity. **a** Cell morphology images captured using TEM at three different increasing magnifications in panels from left to right. **A–C** MCF7 cells treated with control vehicle or **D–F** 1 µM NB73 for 24 h or **G–I** NB73-resistant MCF7 cells maintained in 5 µM NB73. **b** Violin plots in panels from left to right showing mitochondria number per cell, area per mitochondrion, and mitochondria aspect ratio (length/width) in the different groups. Data are expressed as mean ± SEM. *p < 0.05, **p < 0.01. Scale bars are 1 µm (left panels **A, D, G**), 600 nm (middle panels **B, E, H**), and 100 nm (right panels **C, F, I**)

Because cell death is accompanied by changes in mitochondrial morphology, including disruption of cristae and mitochondrial integrity [17, 20], we used transmission electron microscopy (TEM) to examine these aspects in cells sensitive or resistant to FOXM1 inhibition (Fig. 3a, b). These TEM images obtained at three increasing magnifications (going across from the left to right panels) revealed important differences in mitochondria in MCF7 cells grown in medium with control vehicle, versus cells treated for 24 h with NB73, versus cells resistant to killing (NB73R). As shown in the violin plots in panel b, the number of mitochondria per cell was decreased in the NB73-sensitive growth-inhibited cells and the area per mitochondrion increased, consistent with mitochondrial swelling associated with impaired mitochondrial function. Notably, the number of mitochondria per cell and the area per mitochondrion in the NB73R cells returned more to that of the parental Veh cells, in keeping with their ability to survive and be protected from killing by the inhibitor.

**Fig. 4:**
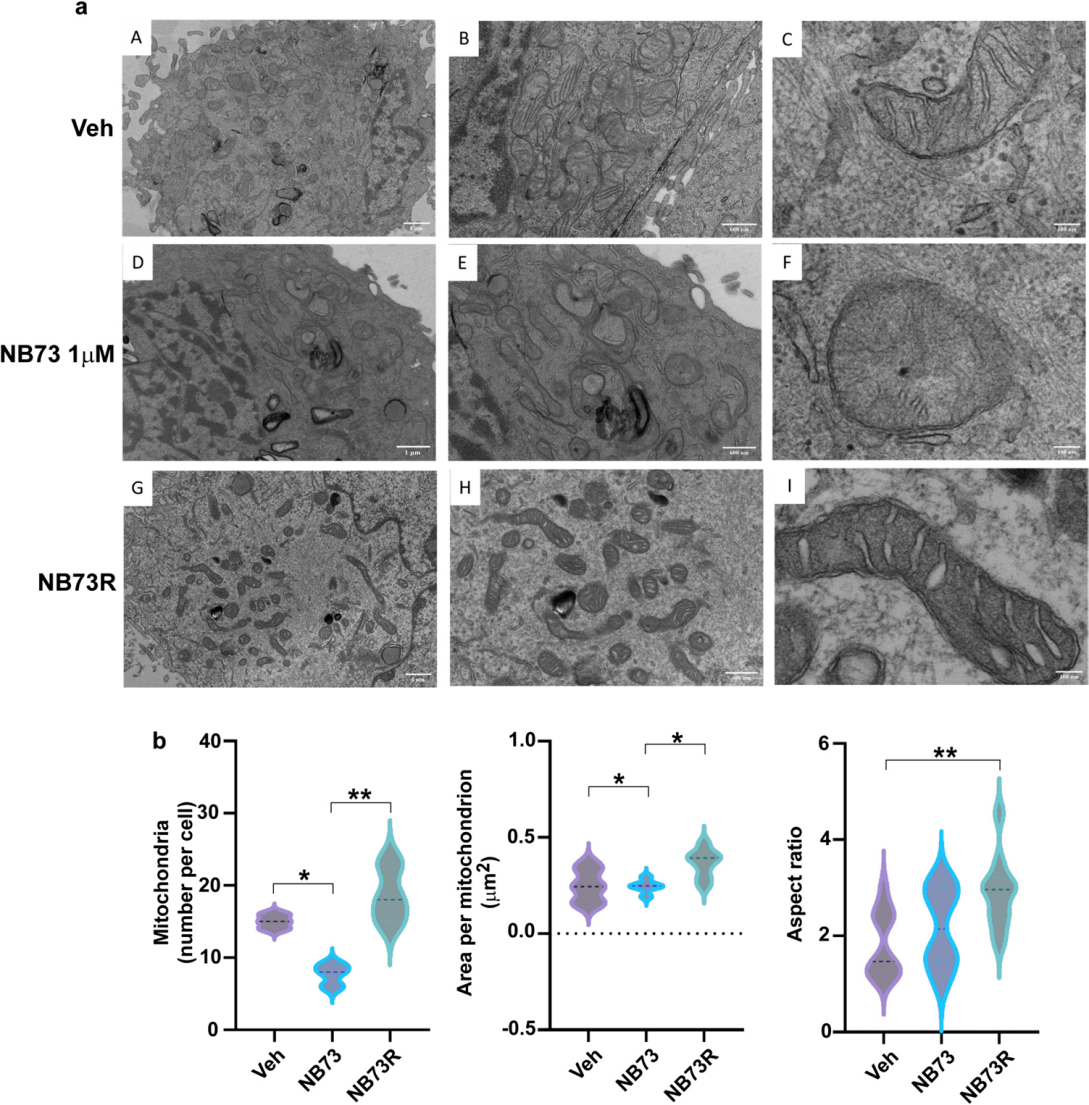
**a** Cell morphology images captured using transmission electron microscopy (TEM) at three different magnifications in panels from left to right. **A–C** MDA-MB-231 cells treated with control vehicle or **D–F** 1 µM NB73 for 24 h or **G–I** NB73-resistant 231 cells maintained in 5 µM NB73. **b** Violin plots showing mitochondria number per cell, area per mitochondrion, and mitochondria aspect ratio (length/width) in the different groups. Data are expressed as mean ± SEM. *p < 0.05, **p < 0.01. Scale bars are 1 µm (left panels **A, D, G**), 600 nm (middle panels **B, E, H**), and 100 nm (right panels **C, F, I**)

Affects on mitochondrial numbers and morphology in MDA-MB-231-sensitive vs. resistant cells were similar to those seen in MCF7-sensitive vs resistant cells (Fig. 4a, b), showing loss of mitochondrial cristae and membranes in short-term treated cells growth suppressed by NB73 (images D, E, and F) and decreased mitochondria number per cell (panel b left). Resistant cells looked more like the parental Veh cells with more normal intact mitochondrial morphology, number per cell, area per mitochondrion, and increase in the mitochondrial aspect ratio (i.e., length/width of mitochondria) (Panel b right, and seen in images I vs C and F).

### Synergy of NB compounds and ferroptosis inducers to strongly inhibit *cancer* cell survival, migration, and colony formation

**Fig. 5:**
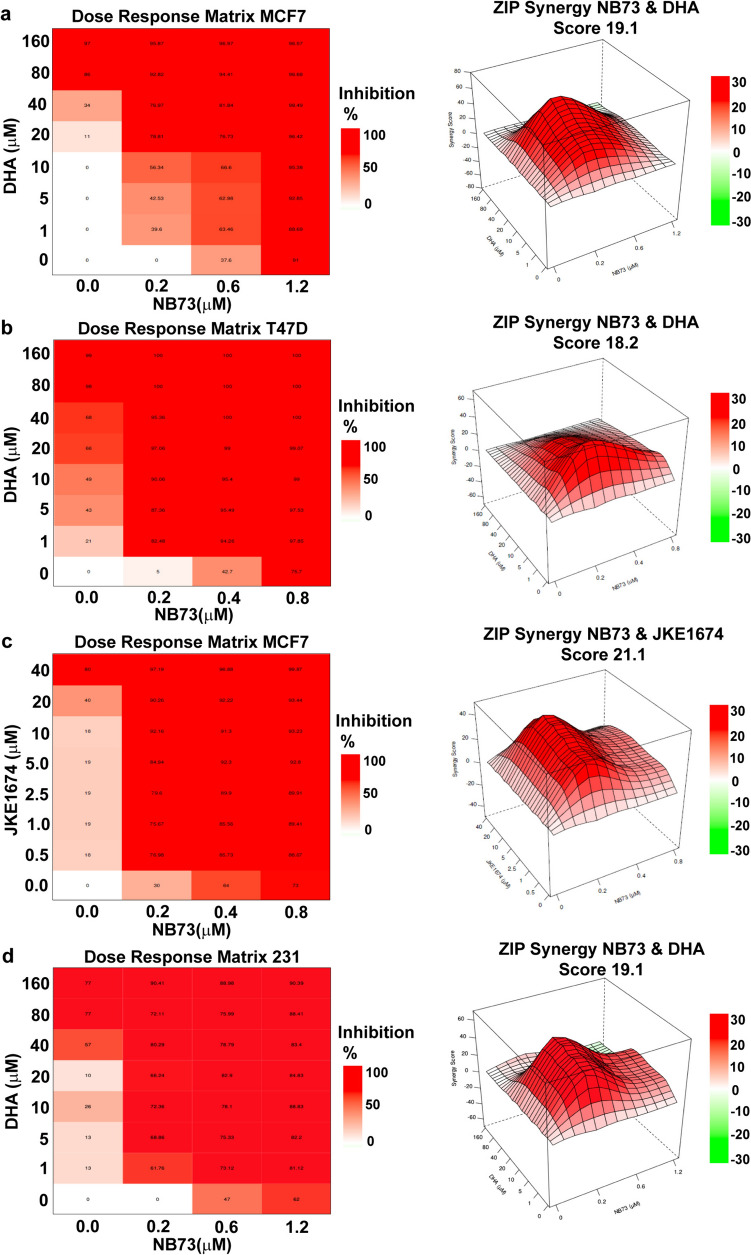
Assessment of synergistic interaction in growth suppression between FOXM1 inhibitor (NB73) and ferroptosis inducer (DHA, JKE1674). Dose response matrix and ZIP synergy score surface plots of NB73 + DHA with **a** WT MCF7, or **b** T47D cells treated with NB73 + DHA, or **c** MCF7 cells treated with NB73 + JKE1674, or **d** MDA-MB-231 cells treated with NB73 + DHA. Cells were treated for 72 h with each compound alone or in combination at each dose pair. An inhibition matrix and ZIP synergy matrix are shown for the combination of compounds. ZIP synergy score is average synergy score of all dose pairs in the matrix with 95% confidence interval, 4 replicates per treatment

The ferroptosis inducers/activators DHA (dihydroartemisinin) and JKE1674 were found to synergize very effectively with low concentrations of NB73 to greatly decrease cell viability (Fig. 5). In these studies, we used a dose–response matrix with two ER-positive breast cancer cell lines (MCF7 and T47D) and treated the cells with NB73 and with DHA or JKE1674 (Fig. 5a–c). We also observed strong synergy between NB73 and DHA in reducing viability of the TNBC MDA-MB-231 cells (Fig. 5d). As shown in Fig. 5, ZIP (zero interaction potency) synergy scores of approximately 20 indicated robust synergy in growth suppression, and that inhibition of FOXM1 with NB73 could effectively sensitize cells to the growth impeding actions of DHA or JKE. Synergy between NB73 and DHA in both ER-positive and TNBC cells suggests that this combination of drugs can work effectively against different subtypes of breast cancer.

**Fig. 6:**
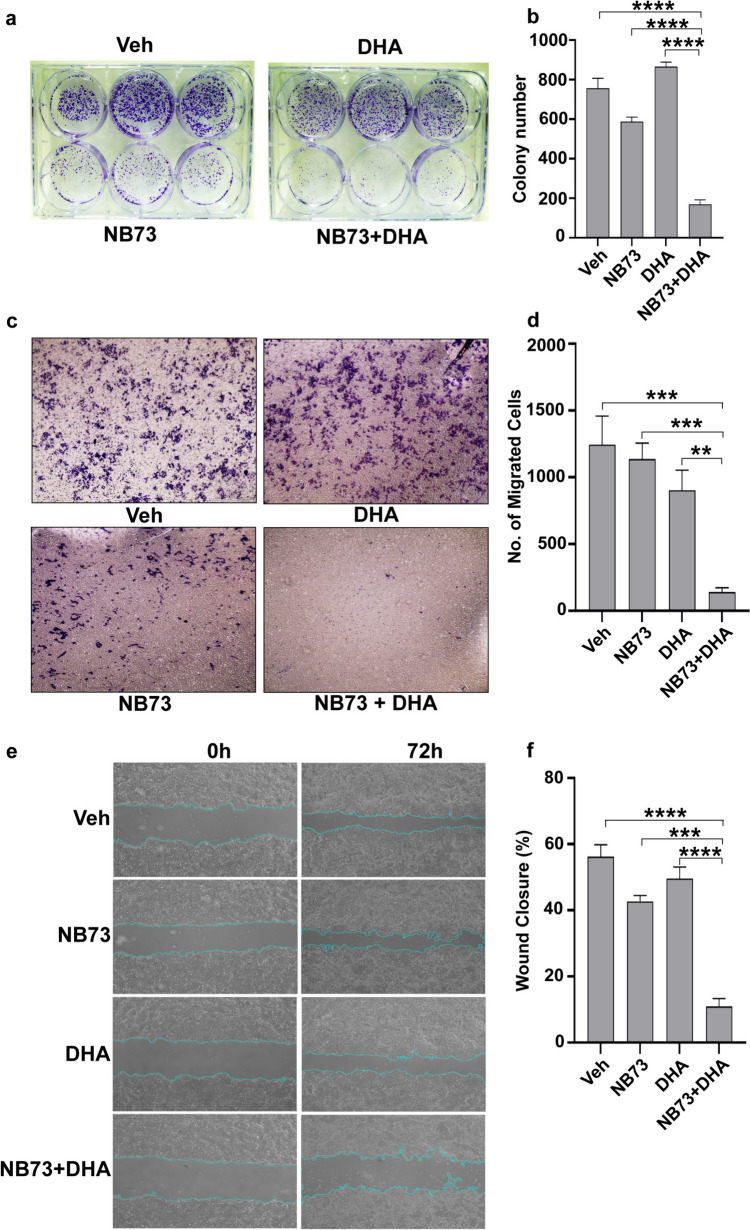
Colony formation and cell migration in cells treated with low concentrations of NB73 or DHA alone or together. **a–b** Effect of NB73 and DHA on colony formation. MCF7 cells exposed to vehicle, or NB73 (0.2 µM), or DHA (1 µM), or NB73 + DHA. Experiments were conducted three times. Images of cell colony plates are shown, and colony numbers were quantitated and are mean ± SEM. ****p < 0.0001. **c–d** MCF7 cells were treated with compounds for 24 h and migration was monitored after 72 h. Data are mean ± SEM; *p < 0.05, **p < 0.01, ***p < 0.001, two-way ANOVA. Data are representative of three independent experiments. **e** Representative images from scratch wound healing assays demonstrating that cell migration into the cell-free region (outlined) is most inhibited with the combination of drugs. **f** Quantitation of percent wound closure at 72 h from 3 independent experiments. Values are mean ± SEM. *p < 0.05; ***p < 0.001; and ****p < 0.0001 for combination versus vehicle or each compound alone

Combined FOXM1 inhibition by NB73 and ferroptosis activation by DHA also synergized to reduce colony formation (Fig. 6a, b) and cell migration, the latter assessed by a two-chamber assay of cell migration (Fig. 6c, d), and a scratch/wound closure assay (Fig. 6e, f).

**Fig. 7:**
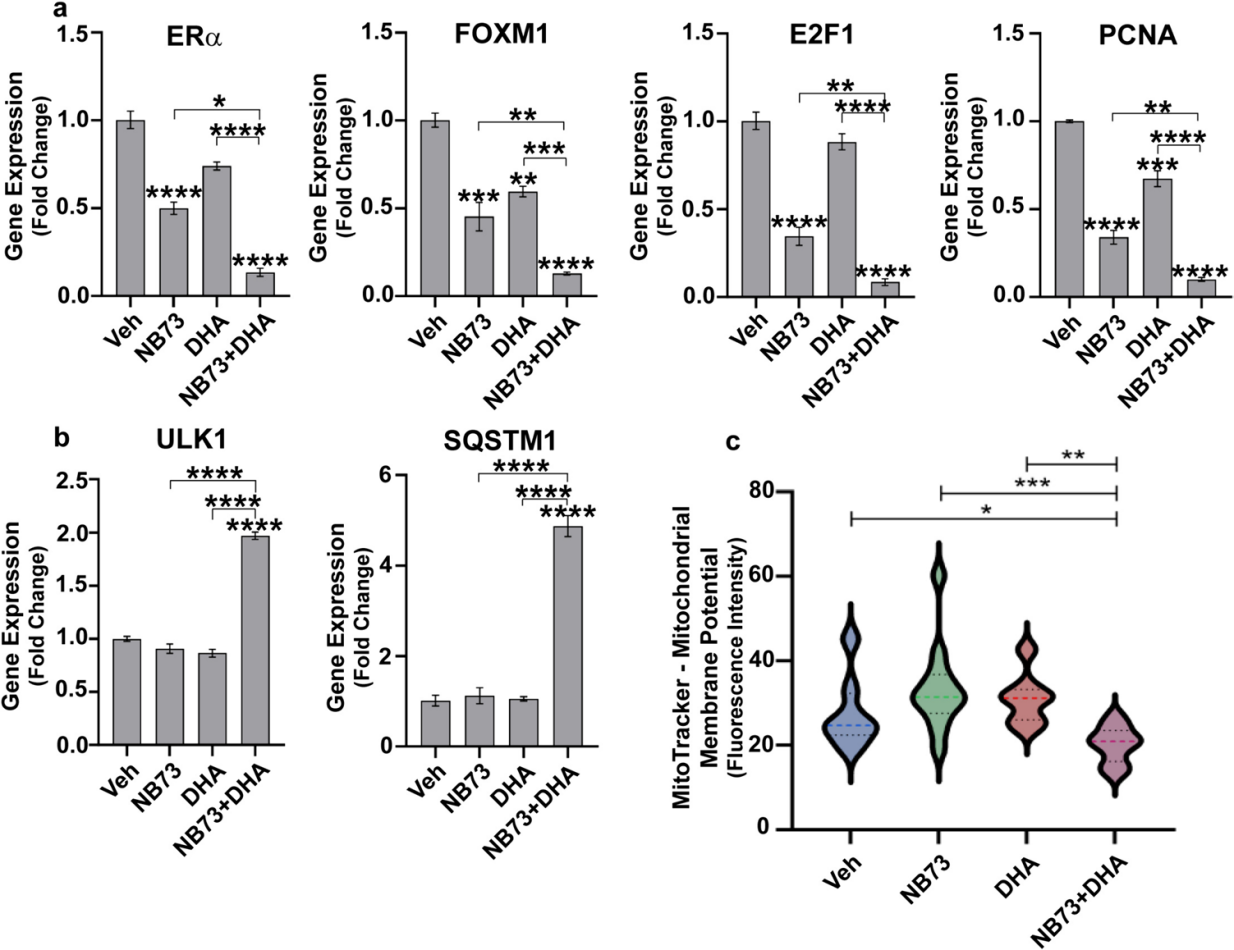
Gene expression and mitochondrial membrane potential of MCF7 cells treated with NB73 or DHA alone or in combination. In panels **a–b**, cells were treated with control vehicle, 0.6 µM NB73, 1 µM DHA, or NB73 and DHA for 48 h prior to isolation of RNA and analysis of gene expression by qRT-PCR. **a** Expression of ERα, FOXM1, and the proliferation-related genes E2F1 and PCNA. **b** Expression of autophagy-related genes. Values are mean ± SEM of 3 independent experiments performed in triplicate. p values are as defined in Fig. 1 legend. **c** MitoTracker assessment of mitochondrial membrane potential in the different treatment groups. Quantitation of fluorescence intensity per cell from live confocal imaging of multiple fields of MitoTracker dye-stained MCF7 cells treated with control vehicle, 0.2 µM NB73, 1 µM DHA, and NB73 plus DHA for 6 h. Data are mean ± SEM of three independent experiments; *p < 0.05, **p < 0.01, ***p < 0.001

The enhanced effectiveness of the combination of NB73 with DHA was also observed in gene regulations (Fig. 7). Low concentrations of NB73 and DHA each decreased expression of ERα and FOXM1, and the proliferation markers E2F1 and PCNA, with combined treatment reducing these much more markedly (Fig. 7a). Likewise, the two autophagy-related genes, ULK1, an initiator of autophagy, and SQSTM1, the autophagy cargo receptor, were only minimally affected by low-dose NB73 or DHA alone, but showed greatly enhanced upregulation with co-treatment, consistent with increased autophagic cell death (Fig. 7b). MitoTracker fluorescence imaging also revealed a reduction in mitochondrial membrane potential in the co-treatment cells, implying impaired mitochondrial integrity and function in cells exposed to both compounds (Fig. 7c).

### Overcoming drug resistance

**Fig. 8:**
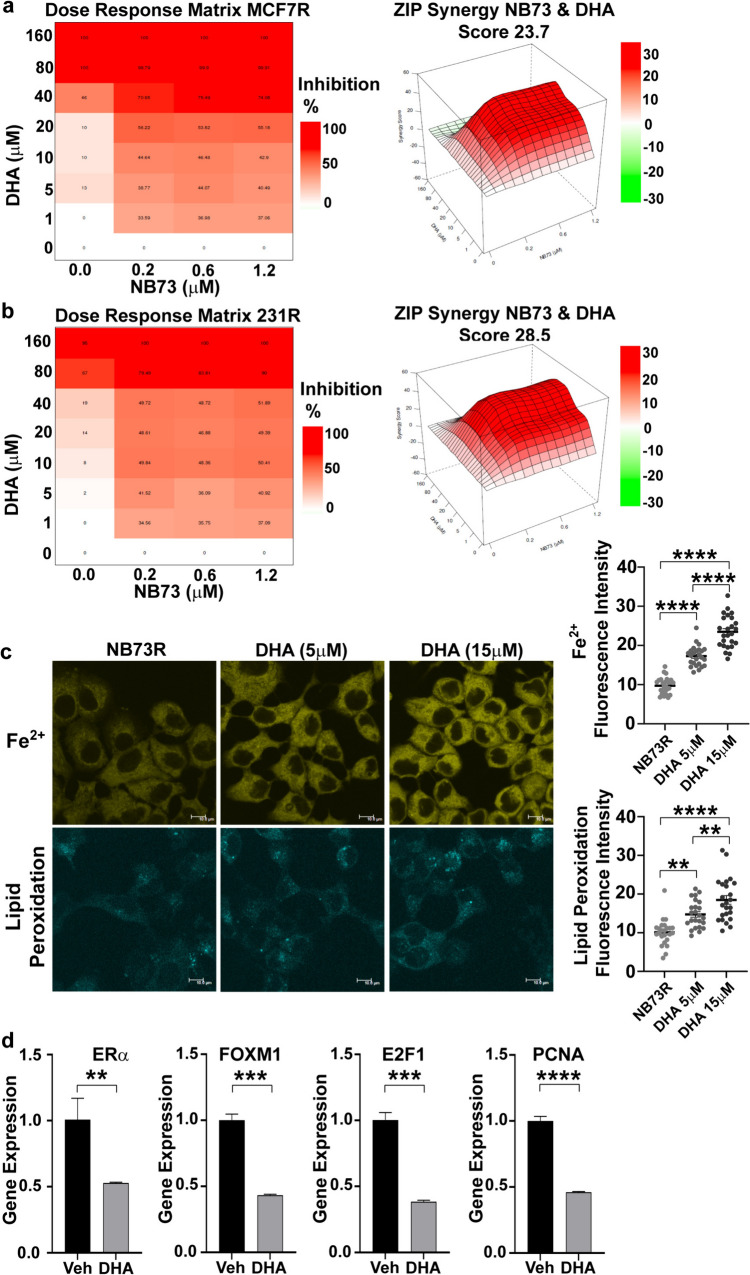
DHA and NB73 work together to inhibit the growth of NB73-resistant cells and affect cell Fe^2+^ and lipid peroxidation and gene expression. **a** MCF7-resistant cells and **b** 231 resistant cells maintained long term in high dose (5 μM) NB73 were changed to cell culture media without NB73 for 24 h. Cells were then treated for 3 days with control vehicle, DHA alone (1–160 μM), NB73 alone (0.2–1.2 μM) or both DHA and NB73 at each dose pair and cell viability was monitored by WST-1 assay. A dose–response inhibition matrix and ZIP synergy score surface plot matrix are shown for the combination of compounds. ZIP synergy score is the average synergy score of all dose pairs in the matrix with 95% confidence interval, 4 replicates per treatment. **c** Cell levels of ferrous ion (Fe^2+^) and lipid peroxidation were monitored in MCF7 NB73-resistant cells maintained in 5 μM NB73 alone and after addition of 5 or 15 μM DHA for 24 h. Fluorescent cell images are shown at the left (scale bar = 10 nm), with quantitation of the fluorescence intensity per cell in multiple fields shown in the graphs at the right. Stars indicate p values of *p < 0.05; **p < 0.01; ***p < 0.001; ****p < 0.0001. **d** Affect of DHA on expression of ERα, FOXM1, and proliferation markers E2F1 and PCNA in MCF7 NB73-resistant cells maintained in 5 μM NB73 and treated with control Vehicle or 20 μM DHA. RNA was harvested at 48 h and gene expression monitored by qRT-PCR. Values are mean ± SEM. Stars indicate p values of *p < 0.05; **p < 0.01; ***p < 0.001, ****p < 0.0001

We next examined whether acquired resistance of the cancer cells to FOXM1 inhibitor could be reversed by a ferroptosis inducer such as DHA. As shown in Fig. 8a,b, the resistant MCF7 and 231 cells, that were not growth suppressed at all by 0.2–1.2 μM NB73 alone, were effectively growth inhibited by 20 μM DHA, a dose that alone was minimally inhibitory (see Suppl. Fig. S1 for full dose-inhibition curves). In addition, intracellular Fe^2+^ and lipid peroxidation levels were low in resistant cells, and DHA dose-dependently increased Fe^2+^ and lipid peroxidation (Fig. 8c). DHA also retained its ability to downregulate FOXM1 and proliferation marker gene expressions (Fig. 8d), as seen in the WT parental cells. These findings indicate that DHA can effectively suppress the viability of resistant cells and could be successfully used in situations where NB73 activity is impaired by resistance.

## Discussion

### Acquired resistance to FOXM1 inhibition

Resistance to cell death is a hallmark of cancer [39], and our studies reveal that in breast cancer cells that acquire resistance to the growth suppressive effects of FOXM1 inhibitor, cell survival is associated with marked changes in modulators of ferroptosis and autophagy that enable cell viability. Ferroptosis and autophagic apoptosis are increasingly appreciated to be interrelated and to play key roles in cancer and drug resistance [15–22]. The involvement of ferroptosis in drug resistance builds upon our prior work documenting increased apoptosis when cells or tumors were treated with NB compounds [10, 13].

FOXM1 is an oncogenic transcription factor that promotes proliferative signaling by regulating the cell cycle, and a high level of FOXM1 in many types of cancers is associated with a more aggressive phenotype and less good patient survival [11–14]. While cancer cells are markedly suppressed by FOXM1 inhibitor treatment, with continued long-term NB compound treatment, there is pressure to develop resistance. This resistance reflects the lineage plasticity of breast cancer cells and progression to a more aggressive therapy-resistant state due, at least in part, to greater dominance of stem-like cell features that promote cell viability and drug resistance. Indeed, we see the enhanced presence of stem-cell markers in the NB-resistant cells. Because we have reported that the FOXM1 gene is not mutated in the inhibitor-resistant cells [13], it suggests that the cell adaptation changes with resistance might be epigenetic with transcriptional rewiring, aspects requiring further study in the future. In this regard it is notable that acquired resistance to endocrine therapies has been reported more recently to include not only somatic alterations but also epigenetic changes with stem-cell promoting factors like FOXM1 underlying the mechanisms of endocrine resistance to agents such as tamoxifen [1, 6, 40].

Because ferroptosis, a key form of regulated cell death, is characterized by lipid peroxidation and involvement of iron, resulting in damage to cell membranes including those of mitochondria, we examined these aspects. We observed that lipid peroxidation and intracellular levels of Fe^2+^ were both greatly reduced in the NB-resistant cells, consistent with these changes facilitating their survival, whereas NB73-sensitive parental WT cells showed NB73 impairment of mitochondrial integrity, including reduction or disappearance of mitochondrial cristae that contribute to mitochondrial dysfunction and cell death. Notably, the resistant cells had elevated levels of factors considered to be suppressors of ferroptosis (e.g., NUPR1, LCN2, GPX4) [41–43], so that the cells survive and show restored mitochondrial integrity and number per cell to that more resembling the parental drug-sensitive cells. Because there are now known to be many interrelationships among cell death pathways, it is interesting that resistance to FOXM1 inhibitor involves thwarting of ferroptosis and autophagic apoptosis.

### Synergy between ferroptosis activation and FOXM1 inhibition

There is clearly a great need to define combination treatments most effective against the drug-resistant cancer cell state, and we have found that promoting ferroptosis by using activators of ferroptosis may be a good way to treat cancer and enhance therapeutic response to FOXM1 inhibition. In fact, our findings have revealed that NB73 sensitizes breast cancer cells to ferroptosis inducers (DHA and JKE1674), suggesting that this combination treatment might hold promise for improving the effectiveness of breast cancer therapy.

The synergistic effects of NB73 and DHA dose combinations in suppressing the viability of parental WT and resistant cells are nicely displayed by the ZIP (Zero Interaction Potential) contours and quantified by their ZIP scores (Figs. 5 and 8). They can also be appreciated from values in the isobologram arrays quantifying the effect that drug combinations have on the IC_50_ values for both DHA and NB73. In both WT and NB73-resistant cells, combined treatments with low doses of NB73 and DHA result in far greater suppression of cell viability than expected from the sum of their activities as individual agents. This synergy was most notable in the NB73-resistant cells, where the IC_50_ value for NB73 dropped more than 25-fold (from > 5 to 0.2 μM) with DHA co-treatment, enhancing NB73 potency in the resistant cells to be equivalent to its potency on WT cells, thereby overcoming NB73 resistance.

Combination treatments are increasingly used in breast cancer therapies, and in general the most effective synergies come from targeting two independent pathways or activities that contribute to the same ultimate outcome of increased cell death. In this study, we utilized compounds that target FOXM1 and an agent that stimulates ferroptosis. Our findings have revealed that their synergistic anticancer effectiveness is associated with inhibition of FOXM1 stimulation of the cell cycle and proliferation, that leads to increased autophagic apoptosis by NB compound combined with ferroptotic cell death increased by DHA and JKE1674.

It is also likely that the synergistic effectiveness of these two compounds might result from additional interactions at other levels, such as their combined downregulation of cellular FOXM1. The anticancer effectiveness of NB compounds alone is thought to result at least in part from their ability to reduce the cellular level of FOXM1 [14]. Notably, in this study, we found that DHA also lowered the cellular FOXM1 level and the expression of proliferation-related genes, suggesting that the combined decrease in FOXM1 by NB73 and DHA might contribute in enhancing their overall inhibitory effectiveness.

It is worth noting that several prior studies have shown anticancer synergies with activators of apoptosis and certain chemotherapy drugs [44] and with endocrine treatment drugs such as tamoxifen [35], and recently with ferroptosis inducers and antiandrogen in prostate cancer [45]. We have also reported synergistic inhibitory efficacy achieved with our NB compounds and proteasome inhibitors in breast cancer and multiple myelomas [8, 9] and with CDK4/6 inhibitors in ER-positive breast cancer [8, 11, 46].

Cell death pathways are increasingly recognized as being interconnected. Ferroptosis regulation is complex and plastic, and is strongly affected by the cellular context [16]. Although ferroptosis is a distinct mode of cell death characterized by iron-dependent lipid peroxidation, there are many interrelationships between ferroptosis and autophagy and apoptosis in control of cell death and cancer therapeutic sensitivity or resistance as shown here.

## Conclusion

The cancer-promoting transcription factor FOXM1 is greatly increased in many cancers and is absent in most normal adult tissues [11]. Cancer cells also have a much higher dependence on iron for their metabolic activity than normal cells. These suggest that utilizing FOXM1 inhibition jointly with activation of ferroptosis might preferentially be directed to killing the cancer while sparing or having only limited effects on normal cells. That NB compound treatment sensitizes breast cancer cells to ferroptosis inducers such as DHA and JKE1674 indicates that their combined use might be efficacious in improving the effectiveness of breast cancer therapy and possibly also the treatment of other types of cancer driven by high levels of FOXM1.

## Supplementary Information

Below is the link to the electronic supplementary material.

Supplementary file1 (PDF 207 KB)
Supplementary Figure S1. Dose response curves showing growth inhibitory effects of DHA or JKE1674 on the viability of WT parental and NB73 resistant MCF7 cells. Cells were treated with control vehicle or the indicated concentrations of DHA or JKE1674 for 72 h and cell numbers were monitored by WST-1 assay. Values are expressed relative to those of the control vehicle cells, and are shown as the mean ± SEM of four determinations. IC_50_ values were determined.

## Data Availability

All data are available within the manuscript or may be obtained from the Corresponding Author (B.S.K) upon reasonable request.

## Abbreviations

DHA: Dihydroartemisinin
ER: Estrogen receptor alpha
NB73: FOXM1 inhibitor
