# Supplementary material for: Resistance to FOXM1 inhibitors in breast cancer is accompanied by impeding ferroptosis and apoptotic cell death
Source: Breast Cancer Res Treat. 2024 Jul 9;208(2):307–20. doi: 10.1007/s10549-024-07420-9 (PMC11455716; doi:10.1007/s10549-024-07420-9)
Supplement: Supplementary file 1 — Supplementary file1 (PDF 207 KB) Supplementary Figure S1. Dose response curves showing growth inhibitory effects of DHA or JKE1674 on the viability of WT parental and NB73 resistant MCF7 cells. Cells were treated with control vehicle or the indicated concentrations of DHA or JKE1674 for 72 h and cell numbers were monitored by WST-1 assay. Values are expressed relative to those of the control vehicle cells, and are shown as the mean ± SEM of four determinations. IC50 values were determined. [file 10549_2024_7420_MOESM1_ESM.pdf]

Resistance to FOXM1 Inhibitors in Breast Cancer is Accompanied by Impeding Ferroptosis and Apoptotic Cell Death

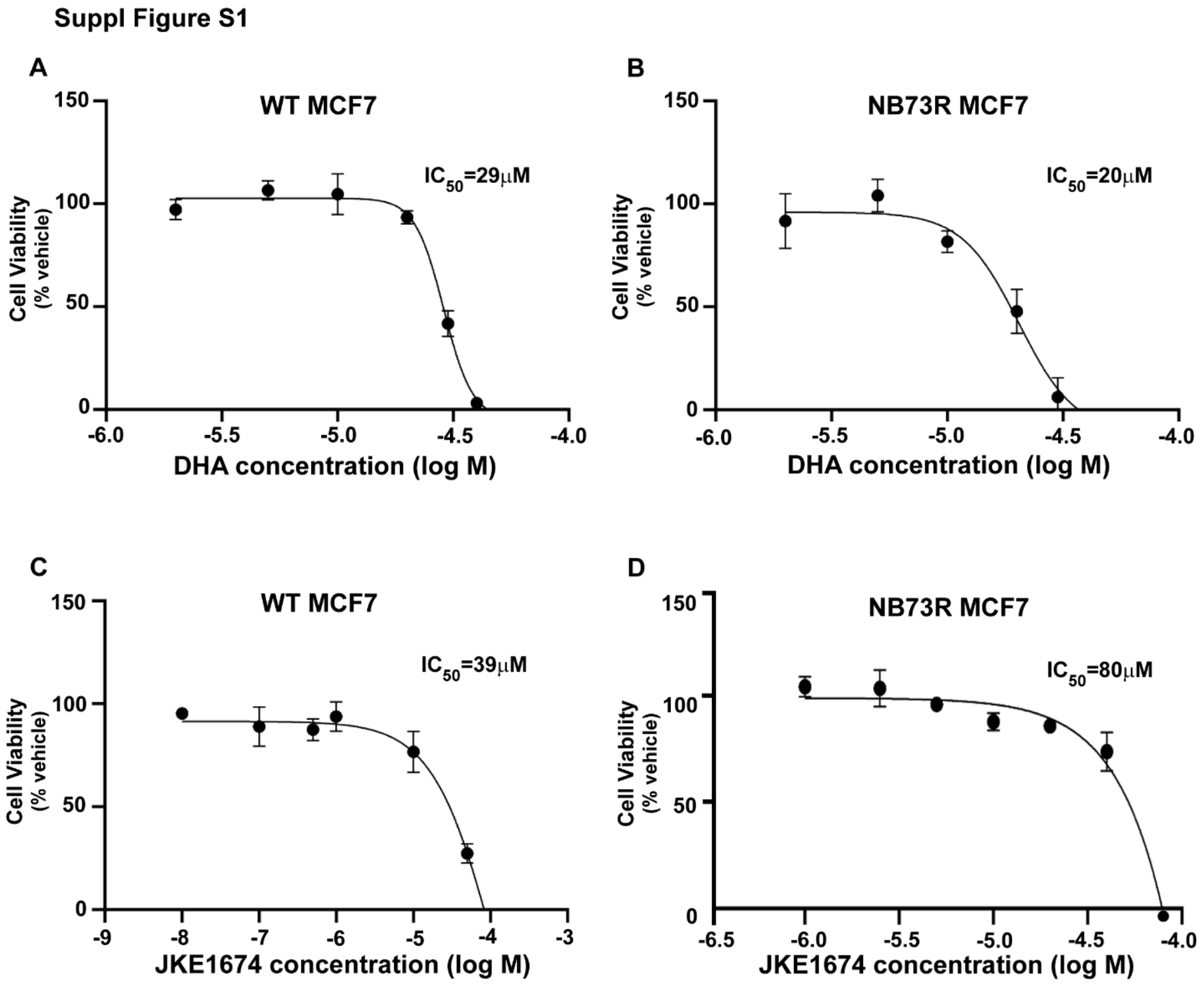

Supplementary Figure S1. Dose response curves showing growth inhibitory effects of DHA or JKE1674 on the viability of WT parental and NB73 resistant MCF7 cells. Cells were treated with control vehicle or the indicated concentrations of DHA or JKE1674 for 72h and cell numbers were monitored by WST-1 assay. Values are expressed relative to those of the control vehicle cells, and are shown as the mean ± SEM of four determinations. IC_{50} values were determined.
